# Responsible Prescribing of Opioids for Chronic Non-Cancer Pain: A Scoping Review

**DOI:** 10.3390/pharmacy8030150

**Published:** 2020-08-20

**Authors:** Eleanor Black, Kok Eng Khor, Apo Demirkol

**Affiliations:** 1 South Eastern Sydney Local Health District, Drug & Alcohol Services, Sydney, NSW 2065, Australia; Apo.Demirkol@health.nsw.gov.au; 2 School of Public Health & Community Medicine, University of New South Wales, Sydney, NSW 2052, Australia; 3 Prince of Wales Hospital, Pain Management Centre, Randwick, Sydney, NSW 2031, Australia; KokEng.Khor@health.nsw.gov.au

**Keywords:** chronic non-cancer pain, opioids, prescribing

## Abstract

Chronic non-cancer pain is common and long-term opioid therapy is frequently used in its management. While opioids can be effective, they are also associated with significant harm and misuse, and clinicians must weigh any expected benefits with potential risks when making decisions around prescribing. This review aimed to summarise controlled trials and systematic reviews that evaluate patient-related, provider-related, and system-related factors supporting responsible opioid prescribing for chronic non-cancer pain. A scoping review methodology was employed, and six databases were searched. Thirteen systematic reviews and nine controlled trials were included for analysis, and clinical guidelines were reviewed to supplement gaps in the literature. The majority of included studies evaluated provider-related factors, including prescribing behaviours and monitoring for misuse. A smaller number of studies evaluated system-level factors such as regulatory measures and models of healthcare delivery. Studies and guidelines emphasise the importance of careful patient selection for opioid therapy, development of a treatment plan, and cautious initiation and dose escalation. Lower doses are associated with reduced risk of harm and can be efficacious, particularly when used in the context of a multimodal interdisciplinary pain management program. Further research is needed around many elements of responsible prescribing, including instruments to monitor for misuse, and the role of policies and programs.

## 1. Introduction

Chronic non-cancer pain (CNCP) is a major public health issue and contributes substantially to the global burden of disease [1,2,3]. Low back pain, migraine, and neck pain affected a combined 1.8 billion people in 2016 [1] and were the first, second, and sixth largest contributors, respectively, to global nonfatal health burden [4]. The global prevalence of CNCP has increased significantly over the past few decades and in some high-income countries, is estimated to affect between 30 and 50 percent of adults [5,6]. This has resulted in dramatic increases in years lost to disability from pain and reduced quality of life [2]. As populations age, these trends are likely to continue, with considerable implications for individuals, health systems, and societies.

Chronic pain is usually defined as pain persisting beyond three months, the period in which tissue healing is expected to occur [7]. CNCP can be nociceptive, neuropathic, or nociplastic and may be caused by a wide range of disease processes including musculoskeletal disorders (e.g., low back pain, osteoarthritis, rheumatoid arthritis), headache, neurological disorders (e.g., diabetic polyneuropathy, postherpetic neuralgia), and fibromyalgia [8]. The underlying mechanism can involve peripheral and/or central sensitisation, and the pain experience may be triggered by an interplay of environmental, cognitive, and emotional processes with nociception or neuropathy [9]. Opioids, which have long been used in treating both acute pain and cancer pain, began to be promoted from the late 20th century as being valuable in relieving CNCP [10]. The rise in CNCP has thus been paralleled by a substantial increase in long-term prescribing of opioids, particularly in the United States, Australia, Canada, and Europe [11,12].

While studies have demonstrated that opioids can provide effective pain relief for many CNCP conditions in the short- to medium-term [13,14,15], their effectiveness with long-term use is less well established [16,17]. Most controlled trials are shorter than 18 months and many are at risk for bias due to nonrepresentative study populations and high rates of discontinuation [18,19]. By contrast, adverse effects related to long-term opioid use are well understood and include disruptions to gastrointestinal, neurological, and endocrine systems [16]. Additional risks associated with prescribed opioids include misuse (including diversion and unsanctioned use) and incident opioid use disorder, the risk for which appears to increase with duration of opioid therapy [20]. CNCP patients receiving long-term opioids frequently have multiple comorbidities that may independently increase their risk for these harms [11]. Fatal and nonfatal overdoses related to prescription opioid use have increased significantly in many developed countries over the past decade [18,21], with prescription opioid deaths reaching epidemic levels in the United States [18].

A growing awareness of the public health imperative to address this issue has led to recent efforts to reduce harms associated with long-term opioid prescribing. While this is a welcome response, it has in some instances led to inflexible interpretation of guidelines and enforced discontinuation of long-term opioids [22], which can increase the risk for overdose and death [12]. Clinicians managing patients with CNCP—including Pain Medicine specialists and General Practitioners, amongst others—face the complex task of safely and effectively managing complex pain (potentially over many years), assessing whether opioids are likely to benefit the patient, and acting to mitigate risks associated with long-term opioid use. Clinicians must now navigate these decisions in an era where cautionary guidance around prescribing has increased, but where the number of CNCP patients receiving long-term opioids has perhaps never been higher. This underscores the need for continued and improved evidence-based guidance to support clinicians in making these decisions.

Existing clinical guidelines, generally based on expert consensus, are an important resource for practitioners but the evidence underpinning their recommendations may not be available or readily accessible. Furthermore, while guidelines are necessary, they are not sufficient to ensure responsible prescribing, which is also affected by additional system-level factors such as practitioner training, models of healthcare delivery, and prescribing or monitoring policies. An assessment of the evidence-base for these factors could inform policymakers who are responsible for regulatory decisions that affect prescribing, as well as healthcare practices making decisions around prescribing policies and models of care. While previous reviews have focused on discrete components of prescribing opioids for CNCP, such as strategies to predict or prevent opioid misuse [23], or the association between prescribed dose and unintentional overdose [24], to the best of our knowledge, this is the first scoping review that focuses on studies relating to multiple elements of responsible prescribing. Nicholson and colleagues [25] undertook a similar narrative review on this topic but this was published nearly two decades ago, and there have been substantial changes to opioid prescribing and management of CNCP since that time.

The objective of this scoping review was thus to identify and summarise high-quality and up-to-date evidence for how clinicians, health practitioners, and policymakers can best ensure responsible prescribing of opioids for CNCP. We define ‘responsible prescribing’ as an approach to care that involves adequate assessment of the patient and their concerns; prescribing for appropriate indications and among carefully selected patients; closely monitoring for effects; and acting to reduce harms associated with the use of that medication. Accordingly, we focused on identifying peer-reviewed literature that addressed the following questions:

- How should clinicians select CNCP patients who are suitable for long-term opioid therapy?
- What opioids should be prescribed, and how?
- What are the best monitoring strategies to assess effectiveness, safety, and misuse for patients receiving long-term opioid therapy?
- What system-level policies or regulations enable or assist responsible prescribing?

## 2. Methodology

**Figure 1:**
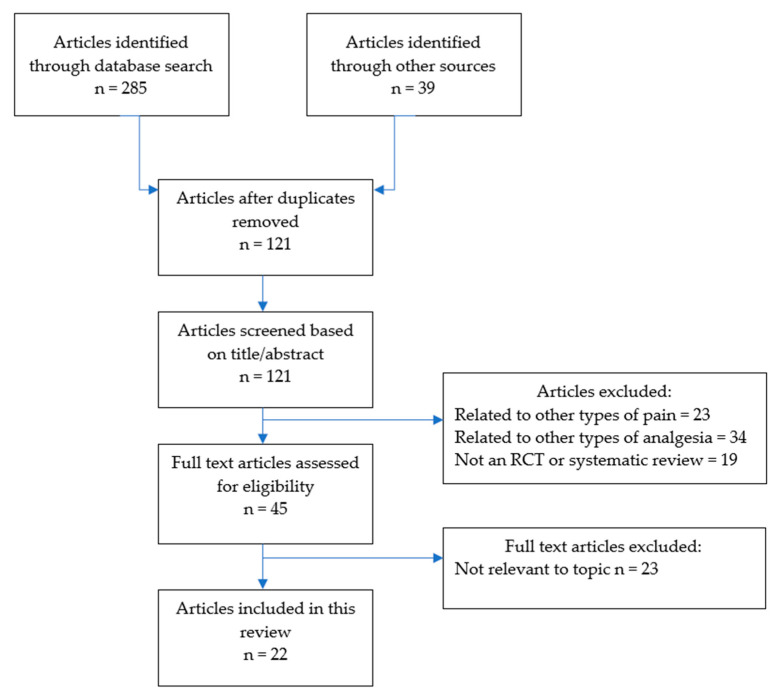
Search strategy.

This scoping review was undertaken in line with the Preferred Reporting Items for Systematic Reviews (PRISMA) guidelines for scoping reviews [26]. The literature search was undertaken over three weeks in April to May 2020. Electronic databases searched included PubMed, EMBASE, OVID, Scopus, Cochrane Database, and Google Scholar, with each being thoroughly searched by combining Boolean logic and truncations for the following key words: “chronic pain”, “opioids”, “chronic non-cancer pain”, and “prescribing”. In addition, the reference lists of included studies were searched by hand. The full search strategy is illustrated in Figure 1.

Identified studies were subjected to the inclusion/exclusion criteria. We excluded studies that were related to chronic pain from malignancy, and studies of CNCP that did not focus on opioid therapy. To be included, studies had to be published from 2005 or later, and a full, English language article needed to be available. We included studies that were either randomised controlled trials (RCTs), or systematic reviews (with or without meta-analyses), and excluded other study types to ensure higher quality evidence. The citations retrieved by the database search were evaluated for inclusion by one of the reviewers (EB) by reading the title and abstract. Full texts of all the publications that could not be excluded at the title/abstract level were read by reviewers. All reviewers screened the abstracts/titles of articles and agreed on which met inclusion/exclusion criteria for full text review. We resolved disagreements on study selection and data extraction by consensus and discussion with other reviewers if needed.

After inclusion, we extracted data using a data extraction form. This collected information on the citation, source, study methods, participants, interventions, outcomes, results, and conclusions. As it was not possible to pool findings from studies, we categorised studies according broadly to their focus, depending on whether they related to patient-related factors; provider-related factors (clinical management); or system-level factors (e.g., regulation or policies).

There were a limited number of RCTs and systematic reviews for some themes, and some of the important considerations for health practitioners involved in prescribing or dispensing opioids for CNCP were not captured by the included studies. Consequently, we made the decision to supplement our findings with a summary of key recommendations from published clinical practice guidelines and position statements developed by the following entities:

- Faculty of Pain Medicine, Australian and New Zealand College of Anaesthetists [27],
- American Pain Society—American Academy of Pain Medicine [28],
- American Society of Interventional Pain Physicians (ASIPP) [29],
- DeGroote National Pain Centre, Canada [30],
- Pain Association of Singapore [19],
- Faculty of Pain Medicine, Royal College of Anaesthetists [31],
- Centers for Disease Control and Prevention [32].

## 3. Results

There were thirteen systematic reviews and nine RCTs meeting inclusion criteria. These covered a range of themes and measured various outcomes, such that a meta-analysis was not possible. The RCTs are summarised in Table 1. There was a weighted geographical focus with all nine RCTs being conducted in the United States. Study duration of the RCTs ranged from 3 days to 13 months. The majority of studies (eight systematic reviews and five RCTs) evaluated prescriber-related factors, such as choice of opioid type, dosing strategies, and mechanisms to improve adherence. There were five systematic reviews and four RCTs that evaluated various system-level factors including guideline adherence, clinician training, and impacts of policies and programs. Only one systematic review addressed patient-related factors, and this study evaluated risk assessment tools to identify patients with a higher likelihood of misusing opioids. The following sections describe key findings from the review, grouped thematically.

### 3.1. Patient-Related Factors

#### 3.1.1. Assessment of the Patient and Their Pain

Although we did not identify any RCTs or systematic reviews describing patient assessment and indications for opioid therapy, there is broad consensus among available guidelines and position statements that patient selection is critical, and that this should involve a comprehensive biopsychosocial assessment and pain review [19,27,28,29,32]. Guidelines agree that some types of pain (for example, headache) are unlikely to respond to opioid therapy, and that opioids should only be trialed for moderate-to-severe pain that has failed all other analgesic modalities and adequate allied health assessments. The Australian and New Zealand College of Anaesthetists (ANZCA) suggest engaging the patient in developing pain self-management skills prior to commencing opioids [27]. Multiple guidelines recommend that clinicians only consider a trial of opioids where the benefits are expected to outweigh risks, and in combination with nonpharmacologic and nonopioid pharmacologic therapy [19,27,28,30,32].

#### 3.1.2. Predicting Risk for Opioid Misuse

Numerous guidelines recommend that in determining which patients are suitable for long-term opioid treatment, clinicians should screen for risk factors for opioid misuse [19,30,32]. This requires having a good understanding of what these risk factors are, and effective methods for assessing risk. We identified one systematic review that evaluated the predictive value of various patient characteristics for opioid misuse among CNCP patients [42]. The strongest predictor found among the six observational studies was a personal history of alcohol or other drug abuse, and variables found to be significant in some studies but not others included male sex, and a history of anxiety disorder or prescription drug misuse. There were important limitations to these findings, including an underrepresentation of women in the studies. 

A large number of screening tools have been developed to identify patients at risk of prescription opioid misuse, and some guidelines [19,29] recommend that clinicians use these prior to consideration of opioid therapy. Such tools include the Revised Screener and Opioid Assessment for Patients with Pain (SOAPP) and the Opioid Risk Tool (ORT). Two systematic reviews that evaluated screening tools [32,42] both found a lack of long-term quality data, and limited evidence that screening for opioid abuse by any instrument will reduce abuse. In their review, Turk and colleagues identified nine observational studies evaluating instruments for screening opioid misuse in CNCP. Most of these had small sample sizes and did not indicate the validity of the instrument, and only two of the instruments (SOAPP; and the Current Opioid Misuse Measure—COMM) assessed all of the psychometric and diagnostic domains outlined [42]. A later systematic review by Dowell and colleagues, undertaken to inform guidelines for prescribing opioids for CNCP in primary care, evaluated five studies and found that evidence for effectiveness was inconsistent for the ORT, and limited for other risk assessment instruments, including the Brief Risk Interview [32].

Overall, there is limited evidence around the predictive value of patient characteristics and screening tools for assessing the risk for opioid misuse. We suggest that clinicians consider these factors when assessing suitability for opioids, but if there are uncertainties around patient suitability, then referral to a specialist centre with expertise in both Pain and Addiction Medicine may be sensible.

#### 3.1.3. Informed Consent

Most guidelines [19,27,28,29,31,32] describe the importance of developing a treatment plan prior to initiation of opioid therapy. This should be developed together by the clinician and patient and outline realistic treatment goals around pain, function, and quality of life. The agreement should also outline a plan for withdrawing treatment if goals are not met. Finally, informed consent involves discussing the potential risks and harms from opioid therapy [19,32]. We did not identify any RCTs or systematic reviews that evaluated the effectiveness of treatment plans on patient outcomes or behaviours, but the development of shared treatment goals is clearly an important element of prescribing responsibly.

### 3.2. Prescriber-Related Factors

#### 3.2.1. Initiating and Titrating Opioid Therapy

Most guidelines recommend a trial of opioid therapy before committing to long-term prescribing for CNCP. American Society of Interventional Pain Physicians (ASIPP) guidelines [29] suggest that opioid therapy be initiated with short-acting formulations and lower doses of less than 40 mg oral morphine equivalent daily dose (oMEDD). The suggested duration of an initial trial ranges between guidelines from 1 to 8 weeks [19,27,29,32], at which time the clinician should evaluate benefits and harms from continued therapy.

We identified one RCT [36] evaluating different approaches to dose escalation among 135 patients with CNCP. Participants in the ‘liberal’ dose escalation group had doses increased if pain relief was reported to be inadequate, while those in the maintenance group had dose increases kept to a minimum, with flexibility to increase if there was clear tolerance or acute injury. Significantly greater rates of opioid increase were seen in the escalation group (80% over 12 months compared with 16% in the stable group), but interestingly, there were no differences between groups in terms of usual pain (measured by visual analogue scale), function, or rates of opioid misuse use—although the dose escalation approach showed a small but significantly larger increase in self-rated pain relief from medications [36].

We identified one systematic review [32] that evaluated the association between prescribed opioid dose and opioid-related mortality, with inclusion of findings from nine different case-control and cohort studies. In one of the included studies, a nested case-control study among CNCP patients, an average daily dose of 200 mg oMEDD or more was found to be strongly associated with a nearly 3-fold increase in the risk of opioid-related mortality relative to an oMEDD of <20 mg [43]. A case-cohort study [44] found that CNCP patients receiving daily doses of >100 mg oMEDD had an adjusted hazard ratio of 7.18 for fatal overdose compared with those receiving <20 mg oMEDD, while a recent case-control study [45] found that among CNCP patients with fatal overdose, the average prescribed dose was significantly higher compared with patients with no history of overdose (98.1 mg oMEDD compared with 47.7 mg oMEDD). Although there was not a clear threshold for fatal overdose in that study, nearly half the overdose cases received a daily dose of >60 mg oMEDD [45]. Overall, the findings in this systematic review demonstrated an association between higher doses and increased overdose risk [32].

Guidelines vary on recommended daily dosing thresholds, but all agree that the lowest effective dose should be prescribed. Many guidelines caution around dose increases above a daily dose of 50–60 mg oMEDD [27,28,32], and some recommend referral to a specialist before increasing the daily dose to more than 90 mg oMEDD [30,32].

#### 3.2.2. Opioid Formulation

While there is no direct evidence from randomised trials to suggest that one type of opioid is superior to others, some guidelines and position statements have issued cautions or recommendations around use of particular opioids for CNCP. ANZCA and the CDC recommend against transdermal fentanyl because of a generally poor misunderstanding of its dosing and absorption properties [27,32]. The CDC recommends against methadone, unless clinicians are very familiar with prescribing it [32]. ANZCA recommends that where a patient is receiving multiple different types of opioids, the prescriber should consolidate these into one opioid formulation only.

There continues to be debate around whether immediate-release (IR) or sustained-release (SR) opioid formulations are more effective, or safer, in treatment. Pedersen and colleagues’ 2014 systematic review [46] looked at studies evaluating the effectiveness and adverse event profile of various long-acting versus short-acting opioid formulations, including oxycodone, dihydrocodeine, tapentadol, and tramadol. They identified six RCTs of varying quality, none of which found significant differences in pain score or use of rescue analgesics between the long-acting and short-acting groups. Three of the RCTs found statistically significant differences around adverse events: one trial found less depression and confusion among patients with osteoarthritis receiving IR versus SR tramadol, and two RCTs found less nausea among patients receiving SR formulations of oxycodone or tramadol. However, none of the trials assessed adverse events systemically with validated instruments or measures of severity [46].

Chou and colleagues’ 2015 systematic review [17] identified 3 fair-quality, open-label trials comparing SR versus IR opioids for titration to stable pain control, but results were inconsistent and difficult to interpret because of differences between groups in dosing protocols and opioid doses. In their 2016 systematic review to inform the CDC Guidelines, Dowell and colleagues identified a cohort study which found that initiation with SR opioids was associated with a greater risk of overdose compared with IR opioids [32]. A number of guidelines recommend using IR formulations rather than SR when commencing opioid therapy for CNCP [30,32]. 

#### 3.2.3. Opioid Rotation

Both the Canadian and the American Pain Society guidelines recommend rotating to alternative opioid formulations when patients have persistent pain and/or problematic adverse effects, although evidence supporting this is fairly weak [28,30]. We identified one RCT [34] that evaluated opioid rotation from full opioid agonists (oxycodone or morphine) to sublingual buprenorphine among chronic pain patients. This study had a crossover design, so that all participants received two doses of sublingual buprenorphine, and—one week later—two doses of active full agonist at 50% of their prescribed total daily dose, allowing direct comparison of opioid withdrawal and analgesic effect in the same patients. The study found no significant difference between groups in self-reported pain score or severity of opioid withdrawal, and concluded that opioid rotation to sublingual buprenorphine from a full opioid agonist can be achieved without an increased risk of withdrawal or loss of pain control. However, the number of participants was small (*N* = 35), and the sample size was too small to allow analysis for participants receiving >160 mg oMEDD. Furthermore, analgesic effect was only measured for up to 12 h after participants received the study drug, prohibiting conclusions around longer term impacts.

#### 3.2.4. Monitoring for Effects and Misuse

Guidelines direct clinicians to periodically monitor risk for opioid-related effectiveness and harms, and to incorporate strategies to identify and mitigate the risk for misuse [27,32]. The “5 As” (analgesia; activity; adverse effects; affects; and aberrant behaviour) describe the components that clinicians should consider when monitoring patients [27]. In addition to taking a history and reviewing the patient, there are various tools or strategies that clinicians might use to assist with monitoring, although the evidence base for many of these is limited.

One option available to clinicians is to periodically use a patient-reported or clinician-led instrument. In their systematic review [47], Becker and colleagues evaluated patient-reported instruments that assess safety, efficacy, and/or misuse in opioid therapy for CNCP. They included 14 studies that developed or validated 9 different instruments including the Prescribed Opioids Difficulties Scale (PODS), the Pain Assessment and Documentation Tool (PADT), and the Prescription Drug Use Questionnaire (PDUQ). Most instruments focused on misuse, with fewer efficacy-related questions across all instruments. Although the psychometric properties of most instruments were statistically significant, there were several potential sources of bias and none of the instruments had been tested in clinical practice. In addition, many took a considerable time to administer and were not considered feasible for time-poor clinicians [47]. These findings suggest that further instrument development and validation is needed.

Another option available to clinicians is to conduct urine drug testing (UDT) which allows for a toxicological assessment of recent substance use. While this may be useful in demonstrating aberrant use or noncompliance, evidence for its effectiveness in reducing opioid misuse is fairly weak. In their systematic review on this topic [48], Starrels and colleagues evaluated the association of treatment agreements and UDTs with opioid misuse outcomes, and identified 11 observational studies in pain clinics and primary care settings. They found that in 4 studies with comparison groups, misuse was modestly reduced after treatment agreements with or without UDT; in the other 7 studies, the proportion of patients with opioid misuse after treatment agreements, UDT, or both varied widely (3 to 43%) [48]. Nevertheless, UDTs may still be helpful in allowing prescribers to identify and respond to aberrant behaviours. Some guidelines [29,32] advise clinicians to request UDTs before starting opioid therapy, and as part of subsequent monitoring. 

A systematic review by Timmerman and colleagues [49] looked at the prevalence and determinants of non-adherence (both over- and under-use) to analgesics among chronic pain patients, and included nine studies that evaluated opioid adherence among CNCP patients specifically. The prevalence of non-adherence varied substantially, with up to 47% of patients estimated to be underusing in one study and 51% estimated to be overusing in another. Determinants of non-adherence varied between studies and included younger age, history of mental health or substance use disorder (SUD), lower educational status, and male sex. One high quality study found that long-acting opioids did not increase the likelihood for non-adherence. Overall, study findings were too varied to draw definitive conclusions [49], and suggest that clinicians should not rely on patient characteristics alone to predict non-adherence. Multicomponent behavioural interventions may be effective in improving compliance, with a small but well-designed RCT [37] finding significantly improved opioid compliance among chronic back pain patients who received brief behavioural interventions (monthly UDTs, compliance checklists, and motivational counselling) compared with patients receiving usual care. 

#### 3.2.5. Opioid Tapering

Several guidelines highlight various reasons that opioids might need to be tapered, including when opioid trial goals are not met [27,28], or where patients receive high daily doses without meeting their pain relief and functional goals [28,30]. For patients experiencing challenges in tapering, some guidelines [27,30] recommend a multidisciplinary program approach to reduction, with involvement from an Addiction Medicine specialist if concerns around dependence emerge during tapering [27]. In the United States, there has been a recent focus on strategies to aid opioid dose reduction and cessation. It is important to highlight that mortality related to opioid use may increase if opioids are forcibly ceased or de-prescribed, and the decision to taper opioids should ideally be made together with the patient, and with appropriate supports in place.

We identified three systematic reviews evaluating opioid tapering among CNCP patients. In their 2017 systematic review [50], Eccleston and colleagues reviewed evidence from five RCTs for various methods to assist with voluntary opioid dose reduction and/or cessation. Based on the limited number of studies, they were unable to find strong evidence for any of the various approaches studied which included acupuncture, cognitive behavioural therapy, and mindfulness [50]. Another systematic review from the same year [51] found low quality evidence suggesting that several different interventions—including interdisciplinary pain programs, buprenorphine-assisted dose reduction, and behavioural interventions—may be effective for reducing opioid dose, and that pain, function, and quality of life may improve with opioid dose reduction. However, study quality was assessed as poor for 51 of the 67 studies, and only 4 studies assessed incident opioid use disorder following discontinuation [51]. Finally, Chou’s systematic review found that evidence around the effectiveness of opioid tapering and different strategies for tapering was limited to small, poor-quality studies, prohibiting conclusions [17].

We identified one RCT [33,46] that compared opioid tapering outcomes in a group of CNCP patients with a 22-week intervention (involving psychiatric consultation and 18 weekly meetings with a physician assistant for motivational interviewing and development of pain self-management skills) to usual care. The intervention group improved significantly more in self-reported pain interference (measured by the Brief Pain Inventory), pain self-efficacy, and prescription opioid problems (measured by the PODS problem scale) at 22 weeks. In addition, the oMEDD was lower at 22 weeks in the intervention group compared with the control group, though this was not statistically significant [33].

#### 3.2.6. Managing CNCP Patients with Opioid Use Disorder

We identified on RCT [35] that evaluated different treatment approaches for CNCP patients with Opioid Use Disorder (OUD). This trial evaluated 12 patients with CNCP and self-reported OUD, and compared treatment outcomes between a group randomised to receive tapering doses of sublingual buprenorphine, and a group randomised to receive stable doses. Over 6 months, participants were more likely to adhere to the opioid replacement protocol than the weaning protocol, and those receiving opioid replacement therapy reported improved pain control and physical functioning after 6 months [35].

The CDC guidelines suggest that where patients receiving long-term opioids for CNCP are identified as having OUD during the course of their treatment, Addiction Medicine specialists should be involved for consideration of treatment with opioid agonists such as buprenorphine or methadone, in combination with behavioural therapies [32]. 

### 3.3. System-Level Factors

#### 3.3.1. Policy Approaches

Regulation and guidance around opioid prescribing can take many forms, and the effectiveness of various interventions are not yet well understood. Beaudoin and colleagues [52] reviewed studies that evaluated the impacts of various prescribing policies on provider-level and patient-level outcomes in the United States and Canada. They included 11 studies that investigated a range of approaches, including legislation around safer operation of pain clinics; placing limits on prescribing of postoperative opioids; and implementation of prescribing guidelines. Their review found some evidence for reductions in opioid prescribing and opioid-related overdoses following guideline implementation at both a state-level and a health facility level, but overall, the evidence was of weak to moderate quality and there was no evidence for reductions in opioid misuse following implementation [52].

Implementing and adhering to guideline recommendations in clinical practice may be challenging, as prescribers may have insufficient skills, resources, or time to do so. Two systematic reviews have evaluated guideline adherence by clinicians. Hossain and colleagues [53] evaluated guideline adherence in 17 cross-sectional studies and 22 chart reviews, encompassing over 11,000 providers in the United States. They found that guideline adherence was generally low, with less than half of prescribers completing treatment agreements, one-third completing UDTs, and just over half assessing for aberrant behaviours. In a similar vein, Tournebize and colleagues [54] undertook a systematic review assessing clinician adherence with opioid risk reduction strategies. Findings from 14 studies were mixed: nearly all physicians considered opioid therapy only when safer approaches had failed, but less than half assessed pain intensity using a scale, and many failed to discontinue opioids if they were ineffective [54]. The authors concluded that guidelines more practical to physicians’ settings, together with further physician training, are needed. 

#### 3.3.2. Prescription Monitoring Programs

Prescription monitoring programs (PMPs) aim to reduce misuse and diversion of controlled substances by monitoring prescription and dispensing data [55]. PMPs thus have the potential to support responsible prescribing, but are not available in many settings and differ widely in terms of program features [56]. We identified only one controlled trial [40] evaluating their impact on prescribing behaviours. This RCT was from the United States, where PMPs have been implemented widely, and included 754 patients who had been identified as filling opioid prescriptions by 3 or more prescribers at 3 or more pharmacies within a 3-month period. Patients were randomly assigned to usual care, or to an intervention model where prescription claims data were sent to their prescribers. The prescribers were also sent a letter outlining prescribing behaviours that reduced risk, such as limiting to a single prescriber and pharmacy. Over the 12-month study period, patients in the intervention group had greater reductions in the number of prescribers (23.98%), dispensing pharmacies (16.28%), and opioid prescriptions (15.25%) compared with the control group [40]. This suggests that enhancing prescriber access to prescription data can improve treatment decisions, although notably, important patient safety outcomes (including overdose) were not measured.

A recent systematic review [55] evaluated the association between PMP implementation and changes in nonfatal and fatal overdoses. It included 17 observational studies from the United States, most of which focused on opioid overdose (a small number investigated overdoses from any drug). This systematic review found low-strength evidence from ten studies for a reduction in fatal overdoses following PMP implementation; conversely, three studies found an increase in heroin overdoses. Program features that were associated with a decrease in overdose deaths included mandatory provider review of data; provider authorisation to access data; and monitoring of non-scheduled drugs. Findings of this systematic review were limited by the small number of studies, high risk of bias, and heterogeneity in outcome measurements.

Overall, evidence around the impact of PMPs on patient safety outcomes is insufficient, and further research is needed to identify features of successful programs. Nevertheless, some guidelines recommend that clinicians review PMP data when starting opioids and periodically throughout treatment [29,32]. 

#### 3.3.3. Health Care Provider Training

While clinical guidelines are a useful resource, clinicians require opportunities to develop familiarity and confidence in applying guideline recommendations, and in improving their skills in assessing and managing patients with CNCP. This may be particularly important for clinicians who do not specialise in pain management but who are frequently involved in the care of CNCP patients, such as general practitioners and hospital residents. An RCT by Sullivan and colleagues [41] found benefits of interactive web-based training in improving knowledge and competence among hospital residents. In this RCT, residents were randomised to complete one of two different training types for management of opioids in CNCP. The first involved an interactive, web-based module focusing on collaborative goal setting, shared decision-making and communication skills; the control group was asked to read compatible practice guidelines. Residents exposed to the interactive training had greater increase in knowledge, and greater self-rated competence in the management of opioids for CNCP patients [41]. After training completion, both groups were less likely to prescribe opioids and were more likely to have patients sign an opioid contract and treatment agreement, suggesting that exposure to guidelines as well as interactive training are beneficial.

#### 3.3.4. Model of Healthcare Delivery

Two RCTs evaluated different models for delivering care for CNCP patients receiving opioids. One evaluated the impact of a multicomponent intervention, ‘Transforming Opioid Prescribing in Primary Care’, on adherence to opioid prescribing guidelines and risk for opioid misuse [38]. This was a cluster RCT involving 53 primary care clinicians and their 985 patients at 4 socioeconomically diverse community health clinics in the United States. Both groups had access to an electronic decision-making tool for safer opioid prescribing, which included evidence-based tools for assessing opioid misuse risk. In addition, intervention groups received nurse care management (where a registered nurse was involved in assessing patients, collecting UDT samples, checking PMPs on behalf of the clinician, and collaborating with the clinician to develop appropriate management plans); an electronic registry for data management; and supervision for clinicians by an experienced opioid prescriber. Analyses that controlled for substance use and mental health diagnoses were undertaken at 12 months and showed that patients in the intervention group were significantly more likely to have received guideline-concordant care (defined as a documented patient–prescriber treatment agreement plan and at least one UDT per year), and were 1.6 times more likely to have either a 10% dose reduction or opioid treatment discontinuation. However, there was no difference between groups in the likelihood of early prescription refills [38].

Another RCT from the United States [39] compared a telephone-delivered care intervention to the usual model of care in 250 patients with chronic musculoskeletal pain at 5 primary care clinics. Patients in the intervention group received regular automated symptom monitoring (by interactive voice-recorded telephone calls) assessing pain, function, and relief or adverse effects from analgesics. Intervention patients also had a face-to-face assessment with the nurse care manager, after which a treatment plan was developed by a Pain specialist. Subsequent ‘intervention phone calls’ were made every 2 months by the nurse care manager. At 12 months, patients in the intervention group had significantly greater improvement in their pain severity and interference scores (as measured by the Brief Pain Inventory), and were nearly twice as likely to report at least a 30% improvement from their baseline pain score. At the beginning of the trial, one-third of patients in each group received long-term opioid therapy for their pain, and there was no difference in opioid initiation rates between groups, with only 3.6% of participants being initiated during the trial. The median daily oMEDD among patients receiving opioids did not increase during the trial [39]. The results of this study suggest that analgesic therapy can be optimised via collaborative telecare management; and that significant improvements in pain and function can be achieved using this model without increases in opioid use.

## 4. Discussion

Opioids play a role in the management of CNCP for some individuals but have the potential to cause significant harm, underscoring the importance of responsible prescribing. In this scoping review, we identified a modest number of RCTs and systematic reviews evaluating elements of responsible opioid prescribing for CNCP. Given the increasing global burden from CNCP and the scale of prescription opioid use and misuse, this relatively low number of studies signals a need for further, high-quality evidence around this issue. The majority of studies we identified were from the United States, likely reflecting an enhanced focus on responsible opioid prescribing in the context of the current prescription opioid misuse epidemic. While many of those studies produced findings that are relevant for other countries, studies that evaluate the impact of local policies are potentially less applicable to settings with different regulatory frameworks for opioid prescribing. It would be useful for further RCTs to be conducted in other countries with high rates of opioid prescribing for CNCP, including from countries with relatively lower rates of misuse.

Most of the studies in this review focused on prescriber-related factors and there were markedly fewer studies evaluating how clinicians should identify and appraise patient-related factors when making decisions around the suitability of opioids for CNCP. It is generally agreed [19,27,28,30,32] that a trial of opioid therapy should only be considered where benefits are expected to outweigh risks, and after clinicians have screened for opioid misuse—but to date there is limited evidence for the predictive value of available screening tools and patient characteristics that might increase the likelihood for a relative benefit from treatment. Reducing the number of patients who are inappropriately commenced on long-term opioid therapy is a public health priority and additional research is needed to further evaluate these predictors. 

Available evidence clearly demonstrates that lower daily doses of prescribed opioids cause less harm compared with higher doses, and that effective pain and function outcomes can be achieved with a more cautious approach to dose escalation rather than reflexive dose increases as a response to reported continued pain. There is also some evidence for the benefits of multicomponent behavioural interventions (which may comprise of a mixture of strategies such as motivational counselling, and development of pain self-management skills) in improving treatment compliance and reported pain-relief. Additional evidence is needed around the safety and effectiveness of various opioid formulations, including whether there are particular benefits or risks associated with IR versus SR preparations, or a combination of both. A recent retrospective study [57] found that osteoarthritis patients treated with short-acting opioids had significantly lower opioid equivalent requirements compared with patients receiving longer-acting opioids; further evaluation of this finding through controlled trials would be valuable. In addition, there is a need for further evidence around the predictive value of instruments developed to assist clinicians in monitoring patients receiving long-term opioid therapy. The effectiveness of patient-reported and clinician-led interviews in establishing treatment efficacy and detecting opioid misuse has not yet been demonstrated, while evidence around the impact of UDTs on opioid misuse is mixed.

Studies evaluating the impact of system-level factors on responsible prescribing of opioids were limited, and findings were varied. A small number of studies have evaluated the impact of PMPs following their widespread implementation in the United States. The available evidence suggests that these programs likely result in safer prescribing behaviours including reductions in the number of prescribers and dispensing pharmacies, but can also result in unintended adverse events (including fatal overdoses) if not carefully implemented. Variations in these outcomes depended in part on whether it was mandatory for clinicians to access and document these data. We suggest that PMP data should be used, where available, as they may alert prescribers to aberrant opioid use. However, if patients are identified by these programs, they should not be suddenly cut off from opioids, but rather reviewed and screened for opioid use disorder, to inform a safe management plan.

Two of the studies included in this review reported low health care provider adherence with clinical practice guidelines for the prescription of opioids for CNCP in the United States [53,54]. Adherence was equally low for guideline recommendations with less rigorous evidence (such as UDTs) and recommendations with rigorous evidence (such as optimising nonpharmacological therapies and observing dose thresholds), and a key conclusion of these studies was that providers require further training and education. Poor knowledge and lack of familiarity can certainly result in low adherence, but other factors—such as insufficient resources to implement guideline recommendations—can equally affect provider motivation or capacity to adhere. If clinicians are to follow guidelines that recommend an adequate trial of nonpharmacologic and nonopioid therapy before considering opioids, then health systems approaches are needed that improve access to pain education, pain self-management programs, physical therapies, psychological interventions, nonopioid drugs, social supports, and interventional pain treatments. Guidelines are necessary for supporting safer clinical practices, but are insufficient to drive behaviour changes in clinical practice if not accompanied by supporting health systems and policies.

There have been promising results from newer, interdisciplinary pain programs and models of care in relation to patient outcomes for pain and function, as well as guideline-concordant care. The multicomponent intervention described in Liebschutz and colleagues’ RCT [38] involved use of an electronic registry; nurse care manager; and multidisciplinary collaboration around treatment plans, and was found at 12 months to significantly increase the likelihood of guideline-concordant care. There have also been positive outcomes demonstrated with a telehealth model of care [56], an approach that could have substantial benefits for people whose mobility is affected by pain, or who live in more remote areas. Such models of care have the potential to shape responsible opioid prescribing into the future, and merit further research.

## Figures and Tables

**Table 1:** Summary of included Randomised Controlled Trials.

Reference	Participants	Aim	Intervention	Outcome Measures	Findings	Conclusions
Prescriber-related factors						
[33]	35 CNCP patients receiving long-term opioids, recruited from pain clinics and primary care clinics	To evaluate the feasibility and effectiveness of a prescription opioid taper support intervention	22 weeks of opioid taper support, consisting of: psychiatric consultation, opioid dose tapering, and meetings with a physician assistant to learn pain self-management skills (compared with usual care for control group)	Mean daily opioid dose Brief Pain Inventory (BPI) pain severity and interference subscales Prescription Opioid Difficulties Scale Prescription Opioid Misuse Index Pain Self Efficacy Scale Perceived helpfulness of opioid taper support	At 22 weeks: Adjusted oMEDD was lower in the intervention group, difference not statistically significant (adjusted mean difference = −42.9 mg; 95% CI: −92.42, 6.62; *p* = 0.09) Pain severity ratings decreased in both groups, difference between groups not significant The intervention group improved significantly more than control group in reported pain interference, pain self-efficacy, and prescription opioid problems	An opioid taper support intervention was feasible and enabled reductions in prescribed opioid dose without increasing pain intensity or interference
[34]	39 CNCP patients receiving full opioid agonist therapy and confirmed to be opioid dependent by naloxone challenge	To determine whether CNCP patients receiving high-dose full agonist opioid treatment could be safely converted to SL BPN without inducing precipitated withdrawal or resulting in worsening pain	Double-blind, active-controlled crossover RCT: each group randomised to a different order of treatment. Group one received SL BPN 12 h after last dose of full agonist; and then resumed normal dosing of full agonist. One week later they received half dose of full agonist 12 h after last dose full agonist. Group two received these in the reverse order.	Clinical Opiate Withdrawal Scale (COWS) score Self-reported pain scale score	The mean maximum COWS scores were similar, and numerically lower on SL BPN There were no significant differences in pain ratings between treatments	CNCP patients treated with full opioid agonists can be switched to SL BPN at 50% of the full opioid agonist dose without an increased risk of opioid withdrawal or loss of pain control
[35]	12 CNCP patients receiving opioid therapy, with concurrent opioid use disorder and recruited via a pain management program	To compare a BPN tapering /discontinuation protocol with an opioid replacement protocol using steady BPN doses in CNCP patients with opioid use disorder	Participants in the active comparator arm were started on tapering doses of BPN with gradual reductions over 4 months and discontinuation by 4 months; participants in the experimental arm were continued on a steady dose for 6 months	Completion of the treatment protocol Engagement in behavioural therapy Number of days drinking or using licit or illicit drugs Number of positive UDTs Levels of pain and function	None of the 6 participants in the comparator group could successfully complete the tapering protocol; 5 out of the 6 in the experimental arm were able to complete the steady dose protocol. Completion rate difference between groups was significant (*p* = 0.015) Of the 10 participants who completed the 6-month follow-up, 8 reported improved pain control and functioning, and 5 used alcohol and/or illicit drugs	CNCP patients with opioid use disorder are more likely to adhere to an opioid replacement protocol than a weaning protocol; steady doses of BPN are associated with improved pain control and functioning compared with tapered dosing
[36]	135 CNCP patients recruited from a chronic pain clinic	To compare the effectiveness of a liberal versus conservative approach to dose escalation among CNCP patients receiving opioid therapy	Participants in escalating dose group who reported inadequate pain relief were given moderate opioid dose increase; participants in the stable dose group had increases kept to a minimum, and only when medically necessary	Visual analogue scales for pain severity, interference, and relief Addiction Behaviours Checklist (monthly) Occurrence of contract violations resulting in treatment discontinuation	At 12 months: No difference between groups for outcomes of usual pain or functional disability, although there was a small but significantly larger increase in self-rated pain relief in the escalating dose group Nearly 27% participants were discharged during the study due to opioid misuse	The escalating dose strategy led to small improvements in self-reported pain relief without an increase in opioid misuse; no differences between groups for other measures
[37]	42 CNCP patients (back or neck pain) meeting criteria for high-risk for opioid misuse	To determine whether cognitive behavioural counselling improves treatment compliance among CNCP patients at higher risk for prescription opioid misuse	Intervention group participated in a structured experimental compliance treatment consisting of monthly UDT, compliance checklists, and motivational counselling (compared with usual treatment protocols for control group)	Drug Misuse Index (a composite score of the Prescription Drug Use Questionnaire; Addiction Behaviour Checklist; and abnormal UDTs)	At 6 months: Significant difference in Drug Misuse Index score between groups (positive score in 73.7% of control group compared with 26.3% in intervention group; *p* < 0.05)	Compliance training and close monitoring may improve treatment compliance among CNCP patients at high risk for prescription opioid misuse
System-level factors						
[38]	Cluster-randomised trial among 53 primary care clinicians and their 985 CNCP patients receiving long-term opioid therapy	To determine whether a multicomponent intervention improves guideline adherence and/or reduces opioid misuse risk	12 months multicomponent intervention consisting of nurse care management, an electronic registry, and electronic decision tools for safe opioid prescribing (compared with electronic decision tool only for control group)	Guideline-concordant care (defined as a documented patient-provider agreement and at least 1 UDT over 12 months) Early prescription refill rate Opioid dose reduction Opioid treatment discontinuation rate	At 12 months: Intervention group more likely than controls to receive guideline-concordant care (65.9% vs. 37.8%, *p* < 0.001; AOR 6.0, 95% CI 3.6–10.2) Intervention group more likely than controls to have either a 10% dose reduction or opioid treatment discontinuation (AOR 1.6, 95% CI 1.3–2.1, *p* < 0.001) No difference between groups in odds of early refills	A multicomponent intervention led to improved provider adherence to guidelines, and patients were more likely to have a reduction in opioid prescription dose
[39]	250 CNCP patients enrolled from primary care clinics with MSK pain of at least moderate intensity	To determine the effectiveness of a telecare intervention for CNCP patients	Participants in the intervention group received telecare management (automated symptom monitoring coupled with an algorithm-guided stepped care approach to optimising analgesia). This was compared to usual care from the primary care physician.	BPI total score BPI interference and severity scores Treatment satisfaction Use of opioids/other analgesics	At 12 months: Compared with usual care, the intervention group had a 1.02-point lower (95% CI, −1.58 to −0.47) BPI score (3.57 vs. 4.59) Intervention group nearly twice as likely to report at least a 30% improvement in pain score (51.7% vs. 27.1%; relative risk, 1.9 [95% CI, 1.4 to 2.7]) Few patients in either group required opioid dose escalation and there were no between-group differences for this	Telecare collaborative management increased the proportion of primary care patients with improved chronic MSK pain
[40]	754 patients recruited from a care organisation who had filled opioid prescriptions by 3 or more prescribers, at 3 or more pharmacies, within a 3-month period	To evaluate the impact on prescribing practices of providing prescription opioid claims information to prescribers	Prescribers in intervention group received a letter and medication report detailing the multiple prescriptions and suggestions to limit number of dispensing pharmacies, as well as a clinical pharmacist contact. Prescribers in control received a letter detailing national trends in prescription misuse.	Change in: Number of prescribers Number of dispensing pharmacies Number of controlled opioid prescriptions	At 12 months: Intervention group patients had a greater reduction (on average 23.98% greater than controls) in the number of opioid prescribers Intervention group patients had a greater reduction (on average 16.28% greater than controls) in the number of pharmacies filling opioid prescriptions Intervention group patients had a greater reduction (on average 15.25% greater than controls) in the number of prescriptions	Enhancing prescriber access to opioid prescription claims information can facilitate informed treatment decisions and improve patient safety
[41]	213 internal medicine residents from 5 medicine residencies	To determine whether an interactive web-based training improves knowledge and competence around opioid prescribing for CNCP	Intervention group completed an interactive, web-based training (‘COPE’—collaborative opioid prescribing education) with a focus on shared decision-making, collaborative goal setting and careful outcome assessment (compared with exposure to clinical guidelines alone for control group)	Knowledge of the role of opioids in CNCP Self-rated competence in CNCP management and opioid prescribing Physician Satisfaction Questionnaire scores Self-reported frequency of using UDTs and opioid contracts Training satisfaction	At 60 days post-training: Intervention group had greater increase in knowledge (X^2^ = 72.06, *p* < 0.00001) and greater self-rated competence in prescribing opioids for patients with CNCP (X^2^ = 5.17, *p* = 0.02) compared with the control group Both groups had significant improvements in satisfaction in managing CNCP, with superior scores in the intervention group for subscales of training adequacy (X^2^ = 4.94, *p* = 0.026)	Exposure to an interactive web-based training was more effective than exposure to practice guidelines for knowledge and competence in prescribing opioids for CNCP

CNCP = chronic non-cancer pain; BPI = Brief Pain Inventory; SL BPN = sublingual buprenorphine; oMEDD = oral morphine equivalent daily dose; UDT = urine drug test; MSK = musculoskeletal.
